# Isolation and genomic characterization of bacteriophage Luminis, a lytic *Yuavirus* infecting clinical strains of *Pseudomonas aeruginosa*

**DOI:** 10.1128/mra.00586-25

**Published:** 2026-07-31

**Authors:** Aaryan Harshith, Zachary N. Flamholz, Paul L. Bollyky, Jessica C. Sacher

**Affiliations:** 1 Division of Infectious Diseases, Stanford Department of Medicine, Stanford University 196258, Stanford, California, USA; Portland State University, Portland, Oregon, USA

**Keywords:** bacteriophages, bacteriophage therapy, *Pseudomonas aeruginosa*

## Abstract

We report the genome of Luminis, a lytic bacteriophage isolated from wastewater using *Pseudomonas aeruginosa* mPAO1Δpf4Δpf6. Phage Luminis is a *Yuavirus* with 61,617 bp of circularly permuted dsDNA and 64.4% GC content. In addition to reference strains, Luminis plaques on two drug-resistant clinical isolates of *P. aeruginosa*.

## ANNOUNCEMENT

*Pseudomonas aeruginosa* is an opportunistic gram-negative bacterium causing skin, lung, and bone infections worldwide (1, 2). Mounting antimicrobial resistance has made it a target for bacteriophage therapy (3, 4). Here, we report the genome of Luminis, a lytic, tailed *P. aeruginosa* phage.

**Fig 1:**
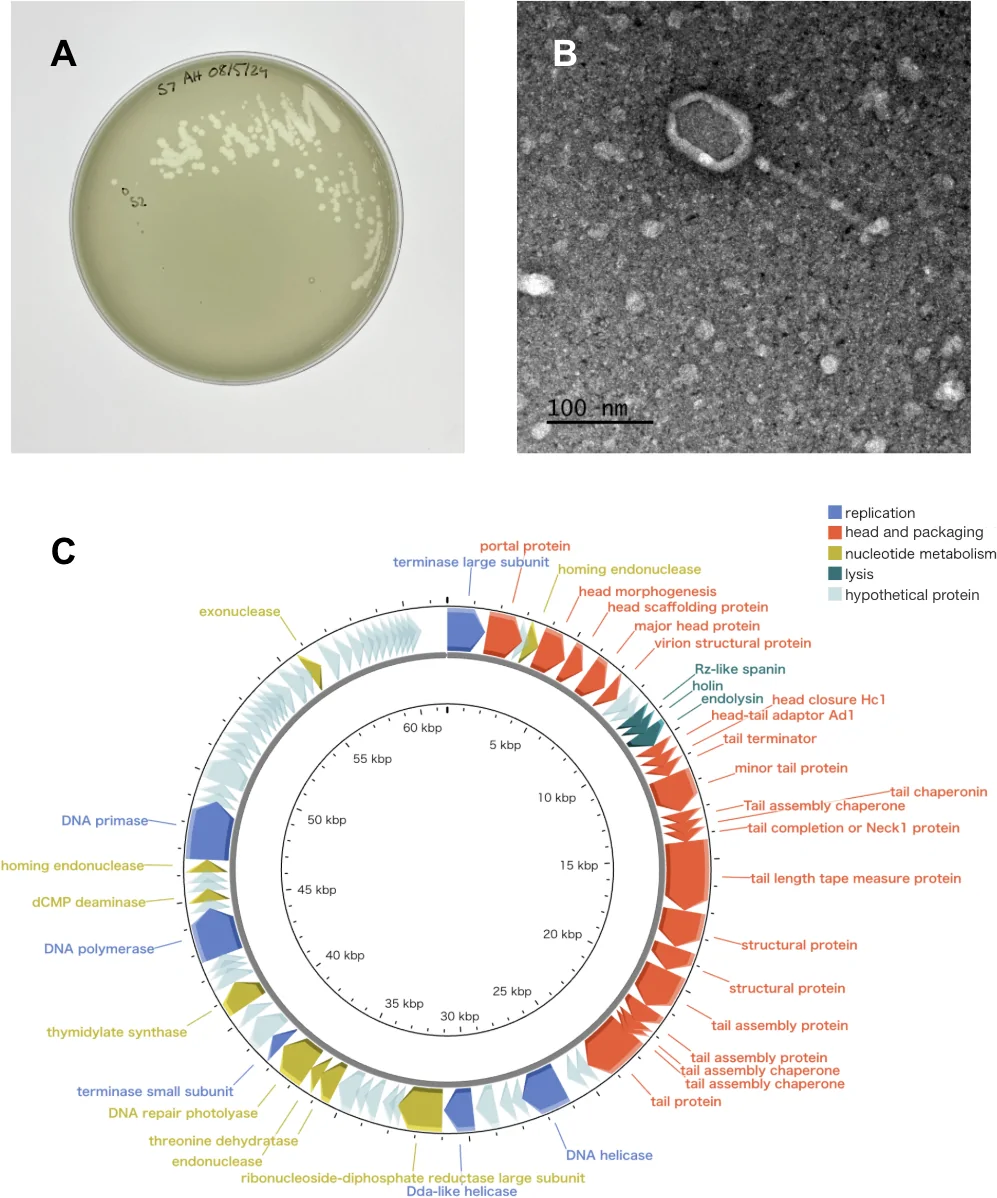
(**A**) Plaques formed by Luminis on *P. aeruginosa* mPAO1Δpf4Δpf6. (**B**) Transmission electron microscopy of Luminis. (**C**) Circularized genome map generated using Proksee 2023 (7). Gene products were categorized by predicted function using Pharokka.

Luminis was isolated from wastewater collected 11 July 2024 at Stanford University’s Codiga Resource Recovery Center. Wastewater was diluted in SM buffer (100 mM NaCl, 8 mM MgSO₄, 50 mM Tris-HCl, 0.01% gelatin), sterile-filtered (0.2 µm polyethersulfone), and enriched for phages using early-log *P. aeruginosa* mPAO1Δpf4Δpf6 (5) grown in LB Miller broth (6) (200 µL wastewater, 5 mL culture, 37°C, 250 rpm). After 4 h, the culture was centrifuged twice (15 min, 5,000× *g*, 4°C). Supernatant (“lysate”) was sterile-filtered and applied to a double-layer mPAO1Δpf4Δpf6 lawn (5). Using a P1000 pipette tip, a round, transparent, 2 mm plaque was transferred into 1 mL SM buffer. To isolate clonal phages, plaque picking was repeated 5× sequentially before propagation (Fig. 1A).

Luminis formed plaques on *Pa* Li010 (8) and two multidrug-resistant (MDR) *Pa* strains from rectal and bronchial infections (CDC AR Isolate Bank AR_0231, AR_0238) (9).

Luminis was negatively stained (1% uranyl acetate) and visualized at 120 kV using a JEM-1400 transmission electron microscope (JEOL, Tokyo, Japan), which showed a flexible, non-contractile tail. Head width, head length, and tail length were 59 ± 2, 81 ± 2, and 139 ± 5 nm, respectively (*n* = 10, mean ± SD, Fig. 1B).

Filtered lysate was treated with DNase (2 U/mL) and RNase A (10 µg/mL) (60 min, 37°C), followed by proteinase K (30 µg/mL, 2 h, 53°C) (New England Biolabs, Ipswich, USA). DNA was extracted using a DNeasy Kit (Qiagen, Germantown, USA). Short-insert Microbial WGS libraries (VAHTS Universal DNA Library Prep Kit for Illumina V3) were prepared and sequenced on an Illumina NovaSeq X Plus Series (PE150) (Novogene, Sacramento, USA).

FastQC (v0.12.1) was used to verify the 9.8 × 10^6^ paired-end reads (150 bp) that were collected (10). Adaptors and low-quality reads (≤100 bp, FastQ ≤20) were extracted with BBDuk (v39.08) (11). Reads were subsampled to 80× with Seqtk (v1.4) (12) and assembled using MEGAHIT (v1.2.9) (13). Contigs were visualized using Bandage (v0.8.1) (14) and validated using BBMap (v38.84) with 100% read coverage (31,912 mapped reads) (15). A single 61,617-bp contig (64.4% GC) was extracted (Fig. 1C) and polished using Pilon (v1.24) (16). PhageTerm (v1.0.12) suggested the genome was circularly permuted and complete (17, 18).

The genome was oriented to the terminase large subunit gene in Geneious Prime (v.2026.0.2) (7, 19) and annotated with Pharokka (v1.8.2) (20). Prodigal (v2.6.3) was used for gene prediction (21), PHROGs for functional annotation (22), and tRNAscan (v2.0.12) for tRNA identification (23). A total of 85 coding sequences were identified, with no tRNAs (Fig. 1C). BACPHLIP (v0.9.6) predicted Luminis was lytic (91% confidence), and Phylogeny.fr (v2.0) classified Luminis into the genus *Yuavirus* (24–26). All tools were run with default parameters unless specified.

## Data Availability

The annotated genome of Luminis is accessible through National Center for Biotechnology Information (NCBI) GenBank (PQ632788). Raw reads are also available (BioProject PRJNA1267730, Sequence Read Archive SRR36204708).
